# rs2569190A>G in *CD14* is Independently Associated with Hypercholesterolemia: A Brief Report

**DOI:** 10.3390/jcdd6040037

**Published:** 2019-10-30

**Authors:** Ali Salami, Christy Costanian, Said El Shamieh

**Affiliations:** 1 Rammal Hassan Rammal Research Laboratory, Physio-toxicity (PhyTox) Research Group, Lebanese University, Faculty of Sciences (V), Nabatieh 1700, Lebanon; a.salami@ul.edu.lb; 2 Faculty of Medicine, University of Ottawa, Ottawa 61350, ON K1G 5Z3, Canada; ccostani@uottawa.ca; 3 School of Medicine, Lebanese American University, Beirut 1102 2801, Lebanon; 4 Department of Medical Laboratory Technology, Faculty of Health Sciences, Beirut Arab University, Beirut 115020, Lebanon

**Keywords:** hypercholesterolemia, *cluster of differentiation 14*, rs2569190A>G, single nucleotide polymorphisms, association analysis

## Abstract

Many studies have assessed the implication of *cluster of differentiation 14* (*CD14*) molecules and its single nucleotide polymorphism rs2569190A>G with different complex diseases, such as diabetes and cardiovascular diseases (CVDs). In this study, we investigated the association of rs2569190A>G in *CD14* with cardiovascular disease risk factors (hypercholesterolemia and hypertension) in 460 individuals from the general Lebanese population (Middle Eastern multiethnic population). Using a multiple logistic regression model adjusted for six covariates (under additive and recessive assumptions), we found that the G allele of rs2569190 in *CD14* was associated with increased levels of total cholesterol (OR = 3.10, *p* = 0.009), low-density lipoprotein cholesterol (OR = 3.87, *p* = 0.003), and decreased levels of high-density lipoprotein cholesterol (OR = 0.38, *p* = 0.001). In contrast, no significant relationship was found with hypertension. Thus, we concluded that rs2569190G in *CD14* is associated with a higher risk of developing hypercholesterolemia.

## 1. Introduction

According to the World Health Organization, cardiovascular diseases (CVDs) represent the most significant cause of death in humans globally [1]. Around 18 million people died because of CVDs in 2016, representing 31% of all deaths worldwide, most of which occur in low- and middle-income countries [1]. In addition to modifiable risk factors such as high cholesterol and triglyceride levels, diabetes, and high blood pressure (BP) levels [2], several studies have revealed an important impact of the innate immune system on the development or the progression of many CVDs [3]. The innate immune system present in multicellular living organisms gives an immediate defense capability against foreign bodies and pathogenic organisms such as viruses, bacteria, and fungi at first exposure [4]. This system depends on the recognition of pathogens by several families of extracellular receptors, such as the cluster of differentiation 14 (*CD14*), which is responsible for triggering innate immune responses and was first identified as marker of monocytes, before being defined as a coreceptor of toll-like receptors [5].

Many studies have assessed the role of *CD14*, especially at the molecular level, and established a link between the gene product and its single nucleotide polymorphisms (SNPs) with different complex diseases such as diabetes [6] and CVDs [7]. For example, the SNP rs2569190 in the promoter region of *CD14* has been found to be implicated in coronary artery disease through changing protein levels [8,9]. Furthermore, the A allele has been reported to be functional and enhances CD14 expression, and thus the host sensitivity, for exogenous or endogenous lipopolysaccharides [10]. More importantly, soluble *CD14* levels have shown strong, independent correlations with traditional cardio-metabolic risk factors and with subclinical measures of vascular disease (carotid wall thickness, ankle-brachial index, and body mass index) [7]. Based on all of the above, we hypothesize that rs2569190 in *CD14* could be associated with CVD risk factors such as hypercholesterolemia and hypertension (HTN). Therefore, the goal of our study was to investigate the association of rs2569190 in *CD14* with CVD risk factors in individuals from the general Lebanese population.

## 2. Materials and Methods

### 2.1. Study Population 

The institutional review board of the Lebanese University approved the recruitment procedure and the genetic protocols (2182/28, on 16 December 2015). This cross-sectional study involved 460 unrelated Lebanese participants who were apparently healthy (free of chronic diseases; cardiovascular or cancer) individuals. 

### 2.2. Clinical and Biological Data Collection

All measurements, including demographic, clinical, and biochemical measurements, were assessed as described previously [11]. Nuclear DNA was extracted from whole-blood samples according to the manufacturer’s recommendations (QIAamp DNA blood mini kit, Qiagen, Hilden, Germany). Very briefly, 4 μg of total DNA from 200 μL of whole human blood was extracted through lysis and continuous spinning. A KASP genotyping assay (LGC group, Berlin, Germany) was used to genotype rs2569190A>G in *CD14*. Hypercholesterolemia was defined as an elevation of total cholesterol (Tchol) and/or low-density cholesterol (LDL-C) levels. Tchol and LDL-C were considered high if their values were ≥150 and ≥100 mg/dL, respectively. High-density cholesterol (HDL-C) levels were considered low if their values were ≤50 and ≤40 mg/dL in females and males, respectively. HTN was defined as systolic blood pressure ≥130 mmHg or diastolic ≥85 mmHg.

### 2.3. Statistical Analyses

SPSS statistical software version 24.0 [12] was used to perform all our statistical analyses except the power analysis. GPower 3.1.9.4 software [13] was used for the power analysis. Continuous variables were presented as mean value ± standard deviation, and categorical ones were shown as numbers and percentages. A chi-squared goodness-of-fit test was performed to determine if the genotypes of rs2569190A>G in *CD14* were in Hardy–Weinberg equilibrium (HWE).

To study the association between rs2569190A>G in *CD14* and hypercholesterolemia and HTN, a multivariate logistic regression model was used while correcting for different confounding factors (age, gender, body mass index, marital status, smoking, and physical activity). This analysis was performed under the assumption of additive (AA vs. GA vs. GG) and recessive models (AA and GA vs. GG). The sample size needed to reach a statistical power of at least 0.90 in a two-sided test with α = 0.05 and an effect size of 0.2 was 409 individuals.

## 3. Results

### 3.1. Characteristics of the Studied Participants

The demographic characteristics of the study participants are shown in Table 1. The group of participants comprised 292 females (63.5%) and 168 males (36.5%). The mean age was 40.6 years old, and approximately 70% of the participants were married (Table 1). In addition, one-fourth of participants were smokers, and only a minority practiced physical exercise once per week (Table 1).

Moreover, the clinical and genetic characteristics are shown in Table 2. Approximately 75% of the participants had high total cholesterol and LDL-C levels, and around half had low HDL-C. 

### 3.2. Association of rs2569190A>G in CD14 with Hypercholesterolemia

Our calculations showed that the minor allele in our population was the allele G, with a frequency of 0.41 (Table 2). In addition, the allelic frequencies were consistent with HWE (*p* = 0.86). Interestingly, the G allele of rs2569190 in *CD14* was associated with increased levels of Tchol (OR = 3.04, *p* = 0.016, Table 3) and LDL-C (OR = 3.83, *p* = 0.006, Table 3) and decreased levels of HDL-C (OR = 0.36, *p* = 0.001, Table 3). Similar results were also seen with a recessive genetic model. Age was positively associated with an increase in Tchol levels (OR = 2.06 and *p* = 0.011, Table 3) and LDL-C levels (OR = 2.22 and *p* = 0.004, Table 3). Similarly, smoking was significantly associated with an increase in Tchol levels (OR = 2.18, *p* = 0.021, Table 3) and LDL-C (OR = 1.88, *p* = 0.050, Table 3) and a decrease in HDL-C (OR = 0.41, *p* = 0.001, Table 3). Participants that practiced physical exercise once per week had decreased Tchol (OR = 0.27 and *p* = 0.001, Table 3), LDL-C (OR = 0.28 and *p* = 0.001, Table 3), and HDL-C (OR = 0.40 and *p* = 0.017, Table 3) levels.

## 4. Discussion

The results of the current study indicated that rs2569190A>G in *CD14* was associated with increased levels of Tchol and LDL-C and decreased levels of HDL-C. This might point to a possible role for *CD14* in the pathophysiology of hypercholesterolemia.

Human *CD14* is located on the long arm of chromosome 5 (q23-31), which encodes the membrane (m)*CD14*, binds to lipopolysaccharides, and activates various TLRs and downstream proinflammatory pathways [14]. Additionally, *CD14* exists in a soluble form (s)*CD14* [15]. In response to interleukin-6, its expression is increased; therefore, it is regarded as an acute phase protein [16]. Of interest, Reiner et al. reported that (s)*CD14* was positively correlated with LDL-C and HTN and negatively correlated with HDL-C [7]. When combined with our results, the above suggests that the *CD14* gene and protein might be implicated in CVDs.

Increasing experimental evidence is suggesting that rs2569190A>G in the *CD14* promoter could contribute to the genetic etiology of human atherosclerosis [17,18]. This SNP was shown to be functional and increase the transcriptional activity of *CD14* [10], which leads to higher (m)*CD14* protein levels and an increased risk of myocardial infarction [17,18].

The current study is not the first to find a link between elements of the innate immune system (*CD14*) and CVD risk factors, since activated innate immune system elements and dysfunction in metabolic pathways could lead to the chronic inflammation and pathologic conditions associated with CVDs [19]. Benachour et al. reported that the expression of the antimicrobial peptide *LL-37* (a component of the immune system) was positively correlated with CVD risk factors such as systolic BP and triglyceride levels, and negatively with plasma levels of HDL-C, in a sample of 90 apparently healthy men [20]. Another component of the innate immune system that has a noteworthy association with CVD risk factors is the human formyl peptide receptor 1 (*FPR1*) [21]. We have previously identified that, in 1012 French middle-aged adults equally divided between healthy and hypertensive individuals, *FPR1* C32T (rs5030878) is associated with increased BP levels [21]. 

It is noteworthy to mention that a Hardy–Weinberg equilibrium for rs2569190 was found despite the participants belonging to the Middle Eastern population, where consanguinity rates are higher than in European populations. This might be explained by the fact that the majority of the studied individuals came from a northern large city (not villages), making mating less consanguineous and more random.

## 5. Conclusions

In conclusion, our results indicate that rs2569190A>G in *CD14* is positively associated with increased Tchol and LDL-C and negatively correlated with HDL-C. This link with hypercholesterolemia might highlight that rs2569190A>G in *CD14* could be implicated in the pathophysiology of hypercholesterolemia. Further studies are needed in order to highlight its possible role in the pathogenesis of CVDs. 

## Figures and Tables

**Table 1:** Demographic characteristics of the study participants.

Characteristics	Participants (*n* = 460)
Age	40.60 ± 14.16
Gender *n* (%)	
Male	168 (36.5)
Female	292 (63.5)
Smoking status *n* (%)	
Nonsmoker	332 (72.2)
Past smoker	6 (1.3)
Current smoker	122 (26.5)
Marital Status *n* (%)	
Single	121 (26.3)
Married	321 (69.8)
Divorced	18 (3.9)
Physical Activity *n* (%)	
<1 per week	345 (75.0)
1 per week	52 (11.3)
≥2 per week	63 (13.7)

Values are arithmetic mean ± SD for continuous variables. Categorical variables are shown as numbers (*n*) and percentages (%). *n*: sample size.

**Table 2:** Clinical and genetic characteristics of the study participants.

Characteristics	Participants (*n* = 460)
BMI (Kg/m^2^)	25.71 ± 4.98
Total cholesterol (mg/dL)	181.41 ± 40.94
High total cholesterol levels *n* (%)	351 (76.3)
LDL-C (mg/dL)	117.39 ± 33.52
High LDL-C levels *n* (%)	347 (75.4)
HDL-C (mg/dL)	45.53 ± 14.61
Low HDL-C levels *n* (%)	270 (58.7)
Triglycerides (mg/dL)	145.96 ± 124.34
High triglycerides levels *n* (%)	174 (37.8)
SBP (mmHg)	132.07 ± 15.89
DBP (mmHg)	67.82 ± 9.12
Hypertension *n* (%)	255 (55.4)
MAF of rs2569190G in *CD14*	0.41
AA *n* (%)	158 (34.3)
GA *n* (%)	224 (48.7)
GG *n* (%)	78 (17.0)

Values are arithmetic mean ± SD for continuous variables. Categorical variables are shown as number (*n*) and percentages (%). *n*: sample size. BMI: body mass index, LDL-C: low-density lipoprotein cholesterol, HDL-C: high-density lipoprotein cholesterol, SBP: systolic blood pressure, DBP: diastolic blood pressure, MAF: minor allele frequency, *CD14*: cluster of differentiation 14.

**Table 3:** Multiple logistic regression analysis with hyperlipidemia and hypertension.

Variables	Total Cholesterol		LDL-Cholesterol		HDL-Cholesterol		Hypertension	
	OR (95% C.I.)	*p*	OR (95% C.I.)	*p*	OR (95% C.I.)	*p*	OR (95% C.I.)	*p*
rs2569190 AA	1		1		1		1	
GA	0.97 (0.57–1.63)	0.900	0.98 (0.59–1.64)	0.939	0.89 (0.56–1.43)	0.636	0.89 (0.56–1.40)	0.607
GG	3.04 (1.23–7.48)	0.016	3.83 (1.48–9.88)	0.006	0.36 (0.19–0.67)	0.001	0.67 (0.36–1.24)	0.203
rs2569190 AA and GA	1		1		1		1	
GG	3.10 (1.33–7.23)	0.009	3.87 (1.58–9.51)	0.003	0.38 (0.22–0.67)	0.001	0.72 (0.41–1.25)	0.186
**Age**								
<40	1		1		1		1	
≥40	2.06 (1.18–3.59)	0.011	2.22 (1.28–3.86)	0.004	1.58 (0.99–2.51)	0.056	0.73 (0.47–1.16)	0.181
**Gender**								
Male	1		1		1		1	
Female	1.21 (0.69–2.12)	0.503	0.98 (0.56–1.71)	0.931	1.17 (0.72–1.90)	0.523	0.46 (0.29–0.75)	0.002
**BMI**								
<25	1		1		1		1	
25–29.9	0.63 (0.34–1.15)	0.132	0.51 (0.28–0.93)	0.027	2.64 (1.50–4.65)	0.001	0.74 (0.44–1.26)	0.267
≥30	1.15 (0.56–2.34)	0.704	1.19 (0.58–2.43)	0.638	1.02 (0.57–1.80)	0.956	1.40 (0.78–2.51)	0.256
**Marital status**								
Single	1		1		1		1	
Married	0.60 (0.31–1.15)	0.124	0.83 (0.44–1.56)	0.566	0.75 (0.43–1.30)	0.306	0.70 (0.41–1.22)	0.206
Divorced	0.76 (0.21–2.76)	0.681	1.06 (0.29–3.83)	0.935	2.06 (0.57–7.47)	0.271	0.33 (0.10–1.07)	0.065
**Smoking status**								
Non smoker	1		1		1		1	
Past smoker	0.71 (0.11–4.62)	0.720	0.72 (0.11–4.72)	0.735	0.63 (0.11–3.67)	0.607	0.17 (0.03–1.13)	0.067
Current smoker	2.18 (1.12–4.21)	0.021	1.88 (0.99–3.53)	0.050	0.41 (0.24–0.68)	0.001	0.74 (0.45–1.22)	0.233
**Physical activity**								
<1 per week	1		1		1		1	
1 per week	0.27 (0.13–0.58)	0.001	0.28 (0.13–0.60)	0.001	0.40 (0.19–0.85)	0.017	0.99 (0.48–2.04)	0.971
≥2 per week	0.69 (0.33–1.41)	0.304	1.27 (0.59–2.74)	0.536	0.57 (0.30–1.11)	0.100	0.90 (0.48–1.70)	0.752

OR: odds ratio, C.I: confidence interval, BMI: body mass index.
